# Can platelet-rich fibrin act as a natural carrier for antibiotics delivery? A proof-of-concept study for oral surgical procedures

**DOI:** 10.1186/s12903-023-02814-5

**Published:** 2023-03-09

**Authors:** Francesco Bennardo, Luca Gallelli, Caterina Palleria, Manuela Colosimo, Leonzio Fortunato, Giovambattista De Sarro, Amerigo Giudice

**Affiliations:** 1 School of Dentistry, Department of Health Sciences, Magna Graecia University of Catanzaro, Viale Europa, 88100 Catanzaro, Italy; 2 Pharmacology Unit, Department of Health Sciences, Magna Graecia University of Catanzaro, Catanzaro, Italy; 3 Microbiology and Virology Unit, Pugliese-Ciaccio Hospital, Catanzaro, Italy

**Keywords:** Antibiotics, Antimicrobial activity, Antimicrobial resistance, Drug delivery, Oral surgery, Platelet-rich fibrin

## Abstract

**Objectives:**

Evaluate the role of platelet-rich fibrin (PRF) as a natural carrier for antibiotics delivery through the analysis of drug release and antimicrobial activity.

**Materials and methods:**

PRF was prepared according to the L-PRF (*leukocyte- and platelet-rich fibrin*) protocol. One tube was used as control (without drug), while an increasing amount of gentamicin (0.25 mg, G1; 0.5 mg, G2; 0.75 mg, G3; 1 mg, G4), linezolid (0.5 mg, L1; 1 mg, L2; 1.5 mg, L3; 2 mg, L4), vancomycin (1.25 mg, V1; 2.5 mg, V2; 3.75 mg, V3; 5 mg, V4) was added to the other tubes. At different times the supernatant was collected and analyzed. Strains of *E. coli, P. aeruginosa, S. mitis, H. influenzae, S. pneumoniae, S. aureus* were used to assess the antimicrobial effect of PRF membranes prepared with the same antibiotics and compared to control PRF.

**Results:**

Vancomycin interfered with PRF formation. Gentamicin and linezolid did not change the physical properties of PRF and were released from membranes in the time intervals examined. The inhibition area analysis showed that control PRF had slight antibacterial activity against all tested microorganisms. Gentamicin-PRF had a massive antibacterial activity against all tested microorganisms. Results were similar for linezolid-PRF, except for its antibacterial activity against *E. coli* and *P. aeruginosa* that was comparable to control PRF.

**Conclusions:**

PRF loaded with antibiotics allowed the release of antimicrobial drugs in an effective concentration. Using PRF loaded with antibiotics after oral surgery may reduce the risk of post-operative infection, replace or enhance systemic antibiotic therapy while preserving the healing properties of PRF. Further studies are needed to prove that PRF loaded with antibiotics represents a topical antibiotic delivery tool for oral surgical procedures.

## Introduction

Antimicrobial resistance (AR) seriously threatens global health with significantly higher morbidity, mortality, and economic burden [1]. The judicious prescribing of antibiotics by healthcare professionals, including dental surgeons, is crucial in stemming the emergence and spread of resistance [2]. Recently, Goff et al. reported that about 60% of dentists declared a correct antibiotic prescription related to dose and time according to guidelines, even if defensive medicine is one of the reasons they prescribed antibiotics [3].

Directly targeting tissues with local drug delivery strategies is a viable approach to reducing unnecessary antimicrobials [4]. Several carriers for topical antibiotic release, such as hydrogels, nanoparticles, and polymers, were tested [5–8]. Autologous products, such as platelets and fibrin, were used as drug delivery systems [9–11]. In particular, autologous platelet concentrates (APCs), studied in medicine and dentistry for regenerative procedures, promote tissue healing by releasing autologous growth factors over time [12–15].

Antibiotics, analgesics, cancer treatments, and other medications that are typically administered intravenously or orally may also be combined with APCs. Given that APCs could reduce the risk of postoperative infections, potential applications as a drug delivery system may be another area of active research. There is no requirement to add growth factors when using APCs as a matrix because they contain growth factors. It is essential to evaluate how a specific drug could be combined with APCs without altering their intrinsic properties and interactions with blood [16].

Among APCs, platelet-rich plasma (PRP) involves multiple centrifugation steps and the use of anticoagulants and activators [17], and platelet-rich fibrin (PRF) belongs to a second-generation that did not require any manipulation after blood collection and a single centrifugation step [18].

Both preparations were used for the preparation of an antibiotic delivery system using autologous blood with different methods of combination between drugs and APCs. Bielecki et al*.* evaluated the antibacterial effect of PRP, documenting that it inhibits the growth of both *S. aureus* and *E. coli* and that antimicrobial effect was enhanced by systemic antibiotic administration before PRP preparation [19, 20]. Polak et al*.* described PRF as a delivery system for antimicrobials: different volumes of metronidazole, clindamycin, or penicillin solutions were directly added to the tubes before blood centrifugation. The authors reported that antibiotic-loaded PRF had a significantly higher antibacterial activity on *Fusobacterium nucleatum* and *Staphylococcus aureus* than control-PRF. Nevertheless, the authors did not investigate the antibiotic release from the drug-loaded PRF [21]. Siawasch et al*.* reported that the addition of antibiotics to blood before centrifugation for PRF preparation did not statistically significant change the release of PDGF-AB, VEGF, TGF-β1, and BMP-2 at each time point evaluated up to 14 days compared to control PRF [22].

The addition of antibiotics to the blood before preparing PRF could benefit local antimicrobial activity in the oral cavity after surgical procedures. For this reason, it is crucial to understand which antibiotics and at which concentrations can be combined with PRF. The present study aimed to evaluate the role of PRF as a local antimicrobial drug delivery system through the analysis of antibiotic release and antimicrobial activity.

## Materials and methods

According to the Declaration of Helsinki on medical protocol and ethics, the regional Ethical Review Board of Central Calabria (reference for the Magna Graecia University of Catanzaro) approved blood collecting for experiments related to PRF (Prot. No. 23-17.01.19). The approval for the overall study protocol was received from the IRB of the School of Dentistry of the Magna Graecia University of Catanzaro. The study was performed in accordance with relevant guidelines and regulations.

### Population and study design

Systemically healthy volunteers were invited to participate in this study. The exclusion criteria were as follows: person under the age of 18; smoking; use of systemic antibiotics in the past six months; alcohol consumption in the last week before blood collection; pregnancy; lactation. Informed consent was obtained from all patients enrolled after being adequately informed of the risks of blood collection.

The study was divided into two parts. Part A was set up to determine the antibiotic release from PRF membranes after direct administration of local antimicrobials to the blood prior to centrifugation, and part B to assess the antimicrobial effect of PRF membranes prepared with the same protocol.

For part A, thirteen tubes of blood were collected from each of the three donors (mean age 26.33 ± 1.53 years) to prepare PRF. One tube was directly placed in the centrifuge, while in the remaining tubes, different antibiotics were injected with a sterile syringe before centrifugation.

For part B, three tubes of blood were collected from each of the six donors (mean age 27.17 ± 2.86 years) to prepare PRF. One tube was directly placed in the centrifuge, while in the remaining tubes, different antibiotics were injected with a sterile syringe before centrifugation.

### Part A: Antibiotic release

#### Platelet-rich fibrin preparation

PRF was prepared according to the L-PRF™ protocol (Intra-Lock, Boca Raton, FL, USA). Briefly, 9 mL autologous venous blood was collected into plastic tubes with a clot activator (Intra-Spin Red Blood Tube, Intra-Lock, Boca Raton, FL, USA). One tube was used as control (without drug), while an increasing amount of different drugs was added to the other tubes (see next section). The tubes were then centrifuged on a fixed-angle centrifuge machine (IntraSpin™, Intra-Lock, Boca Raton, FL, USA) at 2700 rpm (710 g RCF) for 12 min. After centrifugation the red blood cells (RBC) were removed and the PRF membrane was used in the following experiments.

#### Antibiotics

Gentamicin sulfate (Fisiopharma, Salerno, Italy), Linezolid (Fresenius Kabi, Bad Homburg, Germany), and Vancomycin (Pharmatex, Milan, Italy) were used at a dose commonly used in clinical practice: 1 mg/mL, 2 mg/mL, and 5 mg/mL, respectively. Before the tubes' centrifugation, antibiotics were added to the fresh blood at increasing concentrations as described in the Table 1.

**Table 1:** Information concerning antibiotic addition before PRF preparation

	Added volume (mL)	Total antibiotic amount (mg)
Control	–	–
Gentamicin 1 mg/mL		
G1	0.25	0.25
G2	0.5	0.5
G3	0.75	0.75
G4	1	1
Linezolid 2 mg/mL		
L1	0.25	0.5
L2	0.5	1
L3	0.75	1.5
L4	1	2
Vancomycin 5 mg/mL		
V1	0.25	1.25
V2	0.5	2.5
V3	0.75	3.75
V4	1	5

#### PRF characteristics evaluation

To not modify the results of the subsequent experiments, the following non-parametric characteristics were recorded during the procedures: membrane color (yellow or white), consistency (stable, intact membrane; unstable, fragmented membrane), and separation from RBC (if it was necessary or not to separate the PRF membrane from RBC during transfer to the second tube).

#### Quantification of antibiotic release

Membranes were placed in sterile plastic tubes without additives, repeatedly overlaid with 200 μL of PBS, and held at 37 °C in an incubator (95% O_2_/5% CO_2_) to determine the release of antibiotics. Then, at different times (T1, 24 h; T2, 48 h; T3, 72 h, T4, 96 h) supernatant was harvested and replaced with new 200 μL of PBS. Ninety-six hours after the beginning of the study (T4), the membrane was fragmented in a sterile steel bowl filled with 200 μL of PBS and the liquid after filtration (100 µm Cell Strainer, Falcon, Corning, NY, USA), was collected (TF) and analyzed. The amounts of released antibiotics were quantified with a fully automated clinical chemistry analyzer (CDx90, ThermoFisher, Waltham, MA, USA). Measurements were repeated three times, and the lowest value was recorded.

### Part B: Antimicrobial effect

PRF membranes were prepared following the same protocol as for part A, but test tubes were prepared by adding only 0.50 mL of each antibiotic (gentamicin 0.5 mg, linezolid 1 mg).

#### Microbiological evaluation

Strains of *Escherichia coli (ATCC 1100101)*, *Pseudomonas aeruginosa (ATCC 109246)*, *Streptococcus mitis (ATCC NCTC 12261)*, *Haemophilus influenzae (ATCC NCTC 8143)*, *Streptococcus pneumoniae (ATCC NCTC 7465)*, *Staphylococcus aureus (ATCC B-71-1)* were used for this experiment.

Bacterial suspensions were prepared to match the turbidity of a 0.5 McFarland Turbidity Standard (108 colony-forming units [cfu]/mL) according to the Kirby-Bauer method [23]. Columbia agar with 5% of sheep blood (COS, BioMerieux, Marcy-l'Étoile, Lyon, France) were used for *E. coli*, *P. aeruginosa*, *S. mitis*, *S. pneumoniae*, *S. aureus* strains’ isolation. Chocolate agar plates (HAE, BioMerieux, Marcy-l'Étoile, Lyon, France) were used for the isolation of *H. influenzae* strains.

For each bacterial species, plates were prepared for the evaluation of the antibacterial activity of each drug tested. Each PRF membrane was placed, immediately after preparation, on the respective plate using sterile instruments.

The plates were incubated at 37 °C to observe the growth of any colony after 24 h [24]. At the end of the incubation, any growth or inhibition was observed. The plates were photographed to proceed with the measurement of any inhibition area.

**Fig. 1:**
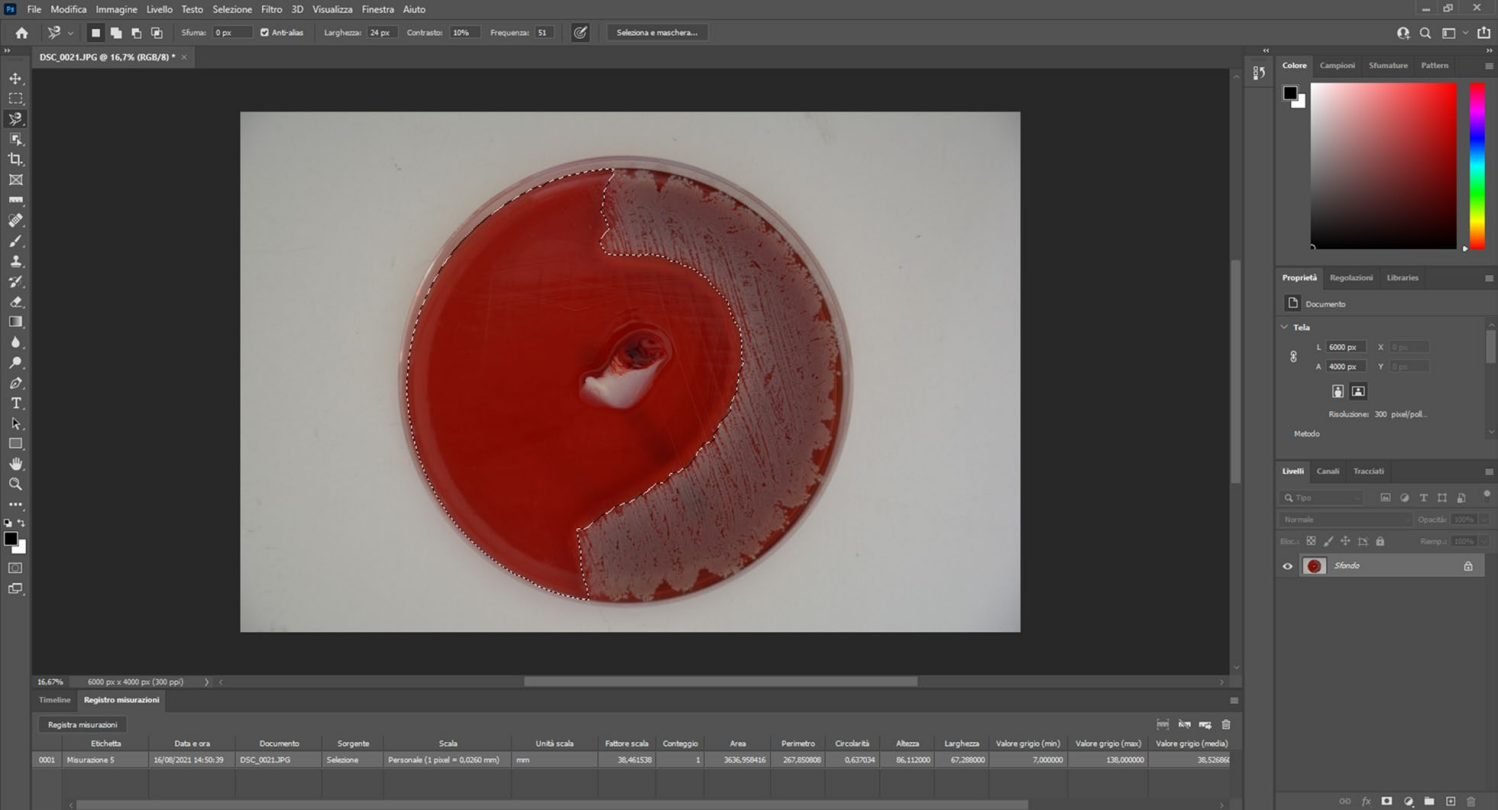
Inhibition area calculation with Adobe Photoshop

The determination of the inhibition area was performed through the software Adobe Photoshop (Adobe Incorporated, San Jose, California, USA): the inhibition area was first outlined using the "magnetic lasso" function, trying to follow as much as possible the color differences within the bacterial growth area; then, a specific unit of measure for each photo was set, selecting the pixels contained in the plate diameter. In this way, each number of pixels corresponding to the known distance set would have had a value of 90 mm. The size of the inhibition area was obtained using Adobe Photoshop calculation function (Fig. 1).

#### Statistical analysis for both parts A and B

Descriptive statistics recorded mean and standard deviation for continuous quantitative variables and absolute and relative frequencies for categorical data. The results were compared using a two-way analysis of variance (ANOVA) test and Tukey's multiple comparisons test to evaluate the main effects of antibiotic quantity and time on drug release. The results were compared using Wilcoxon matched-pairs signed-rank test to evaluate the effects of PRF on bacterial growth or inhibition. P-value < 0.05 was considered significant. Statistical analysis was performed by using GraphPad Prism 9 (GraphPad Prism version 9.2.0, GraphPad Software, San Diego, CA, USA).

## Results

No complications were observed during blood collection in both parts of the study.

### Part A: Antibiotic release

#### Correlation between PRF characteristics and antibiotics

**Fig. 2:**
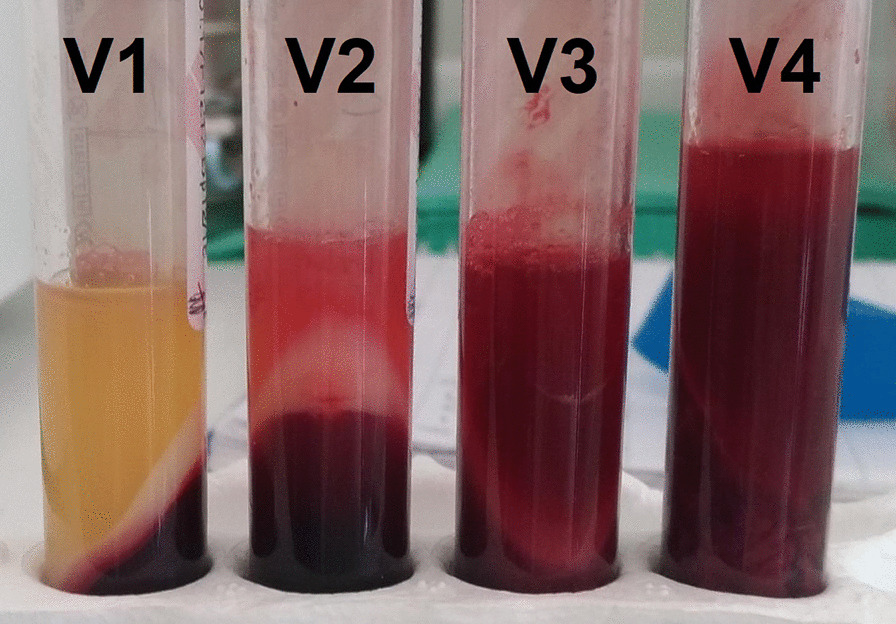
Vancomycin interference in PRF formation

After centrifugation, PRF formation occurred in control tubes. The addition of gentamicin and linezolid in all groups did not change the physical properties of the PRF membranes. Conversely, after the addition of vancomycin, we observed substantial changes in physical properties or no PRF formation (Fig. 2; Table 2). Therefore, vancomycin-PRF was excluded from both parts A and B of the study.

**Table 2:** PRF formation

	Added volume (mL)	Formation	Color	Consistency	Membrane separation from RBC
Control	–	Yes	Yellow	Stable	Separated with scissors
Gentamicin					
	0.25	Yes	Yellow	Stable	Separated with scissors
	0.5	Yes	Yellow	Stable	Almost separated
	0.75	Yes	Yellow	Stable	Almost separated
	1	Yes	Yellow	Stable	Spontaneous separation
Linezolid					
	0.25	Yes	Yellow	Stable	Separated with scissors
	0.5	Yes	Yellow	Stable	Separated with scissors
	0.75	Yes	Yellow	Stable	Almost separated
	1	Yes	Yellow	Stable	Almost separated
Vancomycin					
	0.25	Yes	White	Unstable	Spontaneous separation
	0.5	Yes	White	Unstable	Small piece floating in liquid phase
	0.75	No	–	–	–
	1	No	–	–	–

PRF, platelet-rich fibrin; RBC, red blood cells

#### Release of antibiotics from PRF membranes

**Fig. 3:**
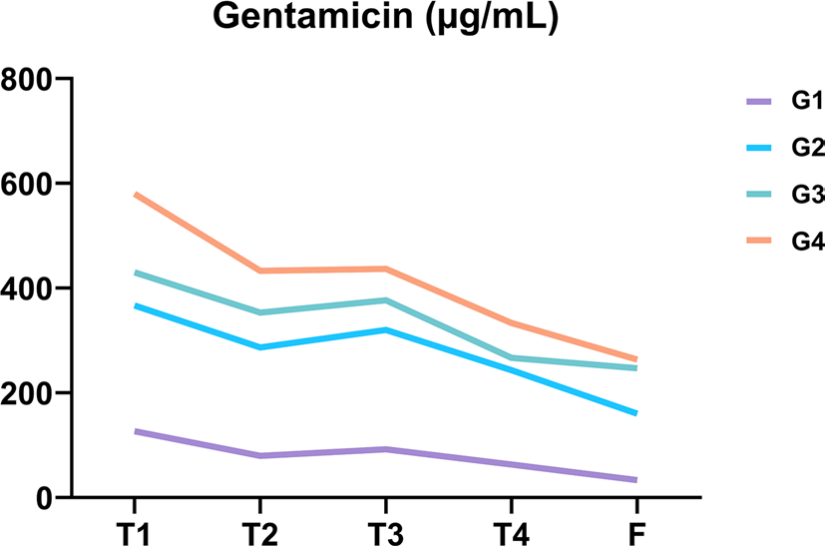
Gentamicin release curve. Data are available in Table 3

**Fig. 4:**
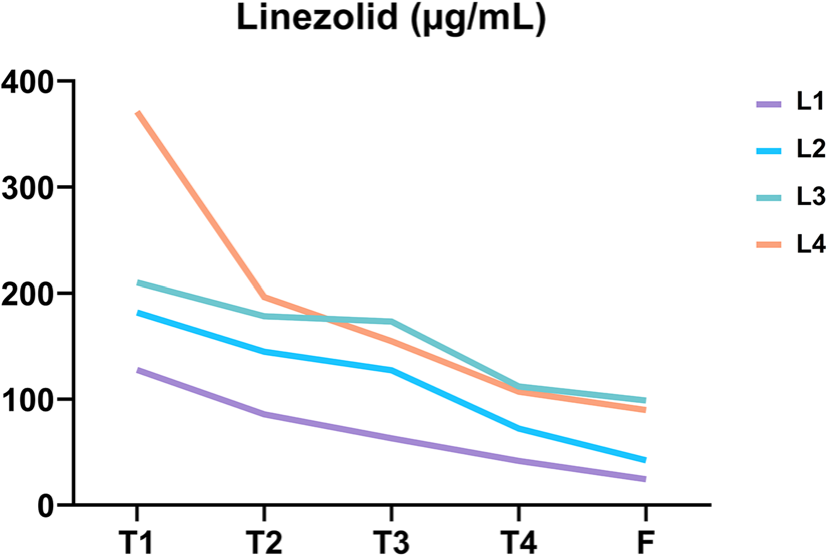
Linezolid release curve. Data are available in Table 4

The analysis of supernatant released from PRF showed that gentamicin (Fig. 3; Table 3) and linezolid (Fig. 4; Table 4) were trapped or bound to the PRF membranes and released over time.

**Table 3:** Gentamicin release analysis (µg/mL; mean ± SD)

	G1	G2	G3	G4
T1	126.7 ± 15.3	366.7 ± 30.6	430.0 ± 43.6	580.0 ± 62.4
T2	79.7 ± 8.3	286.7 ± 20.8	353.3 ± 40.4	433.0 ± 36.1
T3	92.0 ± 6.1	320.0 ± 26.5	376.7 ± 35.1	436.6 ± 25.2
T4	63.0 ± 14.2	243.3 ± 15.3	266.7 ± 30.5	333.3 ± 11.5
F	33.3 ± 1.5	160.0 ± 34.6	246.7 ± 37.9	263.3 ± 41.6

**Table 4:** Linezolid release analysis (µg/mL; mean ± SD)

	L1	L2	L3	L4
T1	127.7 ± 16.6	181.7 ± 21.5	210 ± 26.9	371 ± 30.5
T2	85.7 ± 10.6	144.7 ± 19.5	178.3 ± 18.5	196.3 ± 19.0
T3	63 ± 8.5	127.3 ± 14.8	173.3 ± 16.5	154.7 ± 10.0
T4	41.7 ± 6.5	72.3 ± 8.0	112 ± 12.5	107 ± 7.5
F	24.3 ± 4.5	42.3 ± 3.8	98.7 ± 7.5	89.7 ± 5.9

ANOVA test showed a significant impact of the factors examined (antibiotic quantity, time) on gentamicin release (p < 0.001). Tukey's multiple comparison test within volume groups showed a significant difference between T1 and T2 only for G1 and G2 and between T3 and T4 for G2, G3, and G4. Tukey's multiple comparison test within time groups showed a significant difference only between G1 and G2 at T1, T2, T3, and T4.

ANOVA test showed a significant impact of the factors examined (antibiotic quantity, time) on linezolid release (p < 0.001). Tukey's multiple comparison test within volume groups showed significant difference for all time intervals compared in group A, for all time intervals compared except between T2 and T3 for L2, only between T3 and T4 for L3, for all time intervals compared for L4. Tukey's multiple comparison test within time groups showed significant difference between L1 and L2 at T3, T4 and F, between L2 and L3 at T4 and F, between L3 and L4 at T1.

### Part B: Antimicrobial effect

#### Antibacterial effect of PRF membranes

**Fig. 5:**
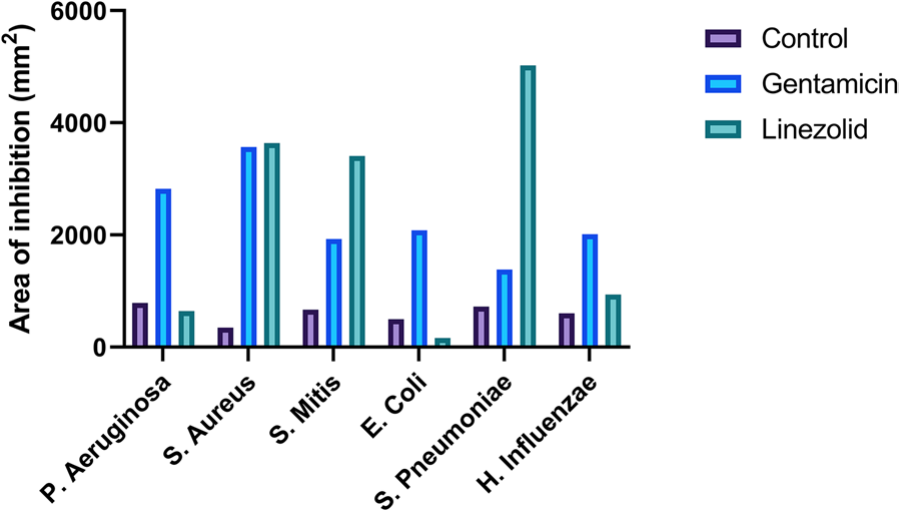
Results of inhibition area calculation. Data are available in Table 5

The inhibition area analysis (Figs. 1 and 5; Table 5) showed that gentamicin-PRF had a massive antibacterial activity against all tested microorganisms. The antibacterial activity of linezolid-PRF was not very effective against *Escherichia coli* and *Pseudomonas aeruginosa*. Control-PRF showed slight antibacterial activity against all tested microorganisms.

**Table 5:** Inhibition area calculated with adobe photoshop

	Strains (highest to lowest inhibition area)	Inhibition area (mm^2^; mean ± SD)
Control-PRF	*Pseudomonas aeruginosa*, *Streptococcus pneumoniae, Streptococcus mitis*, *Haemophilus influenzae*, *Escherichia coli, Staphylococcus aureus*	604.3 ± 160.8
Gentamicin-PRF	*Staphylococcus aureus*, *Pseudomonas aeruginosa*, *Escherichia coli, Streptococcus mitis*, *Haemophilus influenzae*, *Streptococcus pneumoniae*	2300.2 ± 773.2
Linezolid-PRF	*Streptococcus pneumoniae, Staphylococcus aureus*, *Streptococcus mitis*, *Haemophilus influenzae*, *Pseudomonas aeruginosa*, *Escherichia coli*	2301.0 ± 1979.7

PRF, platelet-rich fibrin

Wilcoxon matched-pairs signed-rank test showed that the enhanced antibacterial effect of Gentamicin-PRF compared to control-PRF was statistically significant (p = 0.031). Conversely, the enhanced antibacterial effect of Linezolid-PRF compared to the control-PRF was not statistically significant (p = 0.218).

## Discussion

The aim of this study was to determine if the local addition of antimicrobial drugs had an impact on PRF formation and on its antibacterial activity. For this purpose, the addition of gentamicin, linezolid, and vancomycin to blood prior to centrifugation was investigated. The addition of antibiotics to the PRF produced an antimicrobial preparation that releases drugs in an effective concentration over four days of the experiments, consistent with the first days of healing, with an enhanced antibacterial effect compared to control.

APCs induces an acceleration in the healing of soft and hard tissues, therefore, it can be easily used in periodontology, endodontics, oral surgery, oral medicine, and for the prevention and treatment of osteonecrosis of the jaws [13, 25–31]. In 2018, Miron and Zhang described the possibility of using APCs as a drug delivery system, suggesting its combination with different molecules, including antibiotics [32].

The use of a local delivery system may provide high doses of antibiotics limited to target tissues, exceeding the minimum inhibitory concentration (MIC) up to 1000-fold [33, 34]. Some drugs can both alter wound healing or have cytotoxic effects on various cell types [35]. Other authors used antibiotic solutions (linezolid, gentamicin, and vancomycin) in concentrations commonly used for intravenous administration [36–39]. These authors showed that these antibiotics did not lead to cytotoxic reactions toward cell cultures after seven days of incubation.

Several methods of platelet concentrate drug-loading have been described in the literature: by addition of antibiotics to PRP before coagulation, by a co-delivery applicator, by addition of antibiotics to blood before centrifugation (for PRF), by injection into the PRF membrane after centrifugation [21, 40, 41]. All authors reported successful loading of platelet concentrates with antibiotics.

In agreement with these results, in the present study, we showed that PRF could be prepared with antibiotic loading. Moreover, in our study, the addition of gentamicin and linezolid to blood before the centrifugation did not change the PRF membranes' physical properties with all volumes tested as described in recent literature also for other antibiotics [21, 22]. In contrast, vancomycin addition interfered with PRF formation. Previously, it was reported that vancomycin caused spontaneous RBC aggregation at concentrations > 3.0 mg per ml, and this effect was reversed using sodium citrate, one of the activators used in the production of PRP [42]. This would explain why vancomycin interferes with the PRF formation but not with PRP.

According to previous studies, the release of antimicrobials incorporated in platelet concentrates can be detected for up to one week. Gessmann et al*.* indicated that blood plasma clots could be used to deliver antibiotics, and the antibacterial effects persist for up to five days [36]. Knafl et al*.* reported that teicoplanin and amikacin released from a PRP-antibiotic co-delivery system showed antimicrobial in vitro effects for almost seven days [43]. Wang et al*.* explored the feasibility of using PRP in a local antibiotic delivery system with vancomycin and ceftazidime detecting above 10 times the MIC after 72 h [40]. Siawasch et al*.* reported similar results in the release of metronidazole from PRF membranes after three days [22]. Ercan et al*.* detected the release of doxycycline from drug-loaded PRF in the first 72 h after preparation [41].

Our results are in line with the findings of these researchers. In fact, therapeutic drug monitoring of supernatant obtained during the time of our study (T1-T4) documented a very high concentration of antimicrobial drugs in an effective concentration, upper than the range used in the clinical setting and consistent with the first days of healing. However, the centrifugation protocol used could affect drug concentration, just as it does for platelet concentration [44].

Low-speed centrifugation protocols (A-PRF; A-PRF +) have been introduced as a modification to the original PRF protocol and resulted in modified PRF-matrices with an increased number of platelets, leukocytes, and secreted higher concentrations of growth factors over a 10-day period compared to L-PRF™ [45–47]. Also, a horizontal centrifugation protocol was introduced for PRF preparation resulting in more evenly distributed platelets throughout the membranes when compared to L-PRF™ [48]. Horizontal centrifugation appears to improve the antibacterial properties of PRF, probably due to the increased number of immune cells in the membrane [49].

Not only technical parameters of PRF preparation protocol (RCF value, centrifugation speed, centrifuge, tubes, and time) but also patient gender and age could influence platelet count, antimicrobial efficacy, fibrin network, and growth factors release [44, 50, 51].

Another parameter that should be considered is the effect of resting and compression time post-centrifugation on the characteristics of PRF membranes, which could also affect the antimicrobial effect [52].

Unfortunately, the available literature does not provide clear evidence of the significant clinical advantage of one protocol above the other. Future research is needed to evaluate any changes in antibiotic delivery according to different centrifugation protocols.

In this study, we investigated the effect of drug-loaded PRF on strains of *Escherichia coli*, *Pseudomonas aeruginosa*, *Streptococcus mitis*, *Haemophilus influenzae*, *Streptococcus pneumoniae*, *Staphylococcus aureus*. The inhibition area analysis showed that gentamicin-PRF had a massive antibacterial activity against all tested microorganisms. The antibacterial activity of linezolid-PRF was not very effective against *Escherichia coli* and *Pseudomonas aeruginosa*. Control-PRF showed slight antibacterial activity against all tested microorganisms. Several authors have reported intrinsic antimicrobial activity of platelet concentrates [53]. However, the addition of antibiotics to the preparation seems to increase exponentially the effect [21, 22]. Wang et al. reported that antibiotic-loaded PRP has significantly higher antimicrobial activity against *Staphylococcus aureus*, *Escherichia coli*, and *Pseudomonas aeruginosa* compared to control PRP [40]. Also, Polak et al*.* reported that antibiotic-loaded PRF has significantly higher antimicrobial activity against *Staphylococcus aureus* and *Fusobacterium nucleatum* compared to control PRF [21]. Nevertheless, the results of the microbiological analysis could be influenced by the strain and susceptibility to the type of antibiotic tested [54].

Bielecki et al*.* first reported the possibility of enhancing the antibacterial capacity of platelet concentrates by systemic administration of antibiotics [20]. From a technical point of view, this procedure seems to be easier than antibiotic addition into tubes prior to centrifugation, but the issue of AR should be considered [22].

The results presented in this manuscript proved that the local addition of antibiotics to blood prior to centrifugation resulted in a drug-loaded PRF with significantly higher antibacterial capacity due to the release of antibiotics. This drug delivery system is based on a fibrin network that could bind/embed cells, proteins, and molecules, as described by Miron and Zhang in 2018 [32]. Based on the same mechanism, several authors reported the possibility of combining other drugs with PRF [55, 56].

Antibiotic-loaded PRF, as other systems for local administration of antimicrobials, could increase drug concentration at a specific site with fewer adverse effects compared to systemic administration [57]. Despite local antibiotic delivery is not without issues, despite it represents a therapeutic strategy to combat AR: e.g. a "burst-release" pharmacokinetic profile can have advantages but can also present difficulties in obtaining sustained therapeutic drug levels at the infection site [4, 58]. Nevertheless, local antibiotic therapy may also contribute to AR in long treatment plans [59, 60].

For these reasons, incorporating antibiotics or other drugs into PRF should not be the rule. There are no clinical data to support this drug delivery system, especially in a clinical scenario where PRF could be used for its regenerative and angiogenic capacity. Clinical studies are needed to prove the efficacy of antibiotic-loaded PRF in the treatment of periodontal and peri-implant infections and for the management and prevention of medication-related osteonecrosis of the jaws.

Besides the small sample size, one of the limitations of this study is the lack of analysis of the release of growth factors. Nevertheless, Siawasch et al*.* reported no statistically significant differences in growth factors release between PRF incorporated with antibiotic solution compared to control PRF: for all growth factors (PDGF-AB, VEGF, TGF-β1, BMP-2), a continuous release of up to 14 days was observed in all groups examined [22].

## Conclusion

In conclusion, we documented that PRF could be prepared with antibiotic loading, and the drug is subsequently released from the membrane with an antimicrobial effect. Further in vitro and in vivo studies are needed to prove that PRF loaded with antibiotics represents a topical antibiotic delivery tool for oral surgical procedures that promotes tissue healing and prevents local infection. The type of study represents the main limitation of this study. In particular, the clinical translation of the current in vitro results must be taken with caution as the efficacy of the preparation and any changes in the PRF properties must also be verified in clinical and animal studies. Further studies are needed to evaluate APC use as a drug delivery system for antibiotics and other medications using different preparation protocols. Moreover, this product could reduce the need for systemic drug administration in some clinical scenarios and, consequently, the development of systemic dose-related adverse drug reactions.

## Data Availability

The data that support the findings of this study are available on request from the corresponding author.
